# Association of Dietary Acid Load with Metabolic Syndrome-Related Parameters Following Eating Habit Modification in Korean Adults

**DOI:** 10.3390/nu18050864

**Published:** 2026-03-07

**Authors:** Ye Jin Kim, Chaerin Kim, Jihyun Park, Miok Choi, Won Suk An, Oh Yoen Kim

**Affiliations:** 1 Department of Health Sciences, Graduate School of Dong-A University, Sahagu, Nakdongdaero 550 beon-gil, Busan 49315, Republic of Korea; 1738374@donga.ac.kr (Y.J.K.); 2171722@donga.ac.kr (C.K.); 2277669@donga.ac.kr (J.P.); 2 Department of Food Science and Nutrition, Dong-A University, Sahagu, Nakdongdaero 550 beon-gil, Busan 49315, Republic of Korea; cmo117@damc.or.kr; 3 Department of Nutrition Management, Dong-A University Hospital, 26, Daesinongongwon-ro, Seo-gu, Busan 49201, Republic of Korea; 4 Department of Internal Medicine, Dong-A University College of Medicine, 26, Daesinongongwon-ro, Seo-gu, Busan 49201, Republic of Korea; anws@dau.ac.kr

**Keywords:** dietary acid load, metabolic syndrome, body composition, eating habit modification, nutrition quotient, potential renal acid load, net endogenous acid production

## Abstract

**Background/Objectives:** This study examined the association between dietary acid load (DAL) and metabolic syndrome (MetS)-related parameters in Korean adults undergoing eating habit modification. **Methods:** Forty-eight Korean adults (≥19 years) with at least one MetS risk factor were recruited via public advertisement. Anthropometric and biochemical parameters, Nutrition Quotient (NQ) scores, and nutrient intake were assessed. The DAL was calculated and expressed as the potential renal acid load (PRAL) and the net endogenous acid production (NEAP). **Results:** Forty participants completed the 8-week intervention. Overall improvements were observed in total and domain-specific NQ scores, along with improvements in body composition, blood pressure, and glycemic parameters. Among all participants, the mean DAL scores did not change significantly after FDR correction, although the NEAP showed a modest non-significant decrease. Baseline PRAL and NEAP values did not differ between participants with and without MetS risk improvement. At weeks 4 and 8, DAL indices tended to decrease in the improved group and increase in the non-improved group, with a significant between-group difference observed only for the 8-week change in NEAP after FDR correction. While no significant associations were detected at baseline after FDR adjustment, cross-sectional associations between DAL indices and adiposity-related parameters were observed at week 8, particularly when DAL was expressed as NEAP. However, change-to-change analyses did not remain significant after FDR correction. **Conclusions:** In this exploratory study, DAL levels, especially NEAP, were associated with anthropometric and metabolic status at week 8; however, the absence of significant change-to-change correlations limits causal interpretation. Larger randomized controlled trials are needed to determine whether modification of DAL independently contributes to metabolic improvement (Trial registration number: KCT0011528).

## 1. Introduction

Over the past two decades, the prevalence of metabolic syndrome (MetS) and obesity has increased worldwide, including in Korea [1,2,3,4]. Obesity and MetS contribute substantially to morbidity and mortality, and the proportion of deaths attributable to obesity-related conditions continues to rise in many regions. In Korea, where Westernized dietary patterns have become increasingly common, obesity-related health burdens have also grown considerably [1,2,3,4].

MetS is defined by the presence of three or more of the following components: elevated waist circumference (WC), elevated triglycerides (TG), reduced high-density lipoprotein cholesterol (HDL-C), elevated blood pressure (BP), and elevated fasting glucose [5]. Both obesity and MetS increase the risk of major chronic diseases, including cardiovascular disease, type 2 diabetes, hypertension, certain cancers, and chronic kidney disease [6]. The etiology of obesity and MetS is multifactorial and involves genetic susceptibility, insulin resistance, circadian rhythm disruption, and lifestyle factors such as physical inactivity and unhealthy dietary habits characterized by excessive energy intake [6,7].

Dietary factors, including the increased intake of saturated fat and energy-dense foods, reduced consumption of fruits and vegetables, and adoption of Westernized eating habits, are recognized as major contributors to obesity and MetS risk [8,9]. When such dietary patterns persist, visceral adiposity increases and may alter adipokine and cytokine secretion, thereby promoting insulin resistance, metabolic dysregulation, and chronic low-grade inflammation [8,9]. Consistent with this, previous studies have reported positive associations between Westernized dietary patterns and cardiometabolic risk markers such as the WC, body mass index (BMI), BP, TG, and abdominal obesity [10,11].

In addition to the overall dietary quality, the dietary acid–base balance has recently gained attention as a potential determinant of cardiometabolic health. Diets rich in animal protein, processed foods, and refined grains often accompanied by low fruit and vegetable intake, tend to increase the dietary acid load (DAL), which may adversely influence metabolic homeostasis [12,13,14,15,16,17]. The DAL can be estimated from the nutrient intake and is commonly expressed as the potential renal acid load (PRAL) and the net endogenous acid production (NEAP) [12]. Acid-producing foods typically include meat, fish, eggs, cheese, and refined grains, which provide acid precursors and contribute to higher PRAL and NEAP scores [18]. In contrast, fruits and vegetables are associated with lower PRAL and NEAP scores and may help maintain the acid–base balance [19,20]. Emerging evidence suggests that a higher DAL is associated with obesity-related chronic diseases and adverse cardiometabolic outcomes [20,21,22].

Although the DAL has been extensively examined in relation to obesity, insulin resistance, and MetS components, most previous studies have relied on cross-sectional designs, limiting inference regarding the dynamic responsiveness of DAL indices to dietary modification. Moreover, relatively few intervention-based studies have evaluated whether changes in the DAL are detectable over short-term eating habit modification and whether such changes are metabolically meaningful. Direct comparisons between the NEAP and PRAL in the context of metabolic change also remain limited, and their relative sensitivity and metabolic relevance under lifestyle intervention conditions are not well established. Importantly, evidence is especially limited in Korean populations, where dietary patterns are undergoing rapid transitions toward Westernized eating habits.

Therefore, the present study aimed to explore whether changes in the DAL following short-term eating habit modification are associated with MetS-related anthropometric and biochemical parameters to preliminarily evaluate the relative responsiveness of NEAP and PRAL.

## 2. Materials and Methods

### 2.1. Study Participants and Study Design

Sixty Korean adults (aged ≥ 19 years) presenting at least one risk factor of MetS and without diagnosed chronic diseases were recruited through a public advertisement for this single-arm quasi-experimental pre–post study evaluating eating habit modification. Initially, they underwent baseline screening, at which point, 12 individuals were excluded because they had one or more chronic diseases such as diabetes, cardiovascular disease, stroke, cancer, thyroid, and kidney or liver disease. The remaining 48 individuals were enrolled in the eating habit modification intervention study for 8 weeks. They were asked to visit the study laboratory after 4 and 8 weeks for follow-up for anthropometric measurements (0, 4, and 8 weeks), biochemical tests (0 and 8 weeks), questionnaire surveys (basic information, 24 h recall record sheets, and nutrition quotient (NQ) at 0, 4, and 8 weeks), and eating habit education. During the intervention, eight participants dropped out of the study because of personal reasons (i.e., work time and travel). Overall, 40 participants were included in the final analyses. Among them, 35 (81.4%) were female, 2 (4.7%) were current smokers, and 26 (60.5%) were current drinkers. The Institutional Review Board of Dong-A University approved the study protocol (no. 2-1040709-AB-N-202103-HR-016-04, Trial registration number: KCT0011528). To obtain informed consent, the objectives and contents of the study were explained to the participants.

### 2.2. Definition of MetS

The participants were considered to have MetS when they had three or more of the five factors listed in the NCEP-ATP III guidelines: (1) WC criteria for abdominal obesity proposed by the Korean Society for the Study of Obesity ≥90 cm for men and ≥85 cm for women, (2) TG level of ≥150 mg/dL, (3) HDL-C level of <40 mg/dL for men and <50 mg/dL for women, (4) BP of ≥130/85 mmHg, and (5) fasting blood glucose level of ≥100 mg/dL or a history of taking antidiabetic, antihypertensive, or lipid-lowering medications.

### 2.3. Eating Habit Modification

At the initial visit, the study participants attended a one-hour education session on eating habit modification delivered by professional clinical dietitians and received educational materials and books. The sessions covered the definitions of obesity and MetS and the ways to improve eating habits (i.e., appropriate food choice, reduced consumption of foods high in sodium and saturated fatty acid, guidance on healthy eating-out practices, and food preparation methods). Training for increasing physical activity (light–moderate intensity) in daily life was also provided by health professionals. To increase adherence to the dietary advice, cell phone text messages were also sent to the participants (i.e., low-calorie food selection, stress release, and smart eating-out). This was reinforced by checking their food diary notes.

### 2.4. Calculation of DAL Scores

We encouraged the study participants to maintain a dietary diary for at least 3–6 days each month to estimate the DAL expressed as NEAP and PRAL scores. The NEAP was calculated using protein and potassium intakes, whereas the PRAL was calculated using protein, potassium, phosphorus, magnesium, and calcium intakes [23].

### 2.5. Nutrition Quotient (NQ)

The Nutrition Quotient (NQ), a dietary index developed by the Korean Nutrition Society, was used to evaluate the nutritional status and meal quality of the study participants. It consists of 21 questions divided into four domains (balance, diversity, moderation, and eating behavior) from which the overall NQ score is derived. The balance domain evaluates the adequacy of essential food group intake, including the consumption frequency of fruits, vegetables, dairy products, and protein-rich foods. The diversity domain assesses the variety of foods consumed across and within food groups. The moderation domain evaluates the frequency of intake of foods that should be limited, such as fast foods, fried foods, processed meats, and sugar-sweetened beverages. The eating behavior domain assesses healthy dietary practices, including regular meal patterns, breakfast consumption, and health-conscious food choices. Domain-specific scores and total NQ scores were calculated according to the standardized scoring guidelines.

### 2.6. Anthropometric Measurement and Blood Collection

Each participant’s height was measured manually using an InBody BSM170 extensometer (InBody Co., Ltd., Seoul, Republic of Korea). The body weight, body fat percentage, body fat volume (BFM), visceral fat area (VFA), skeletal muscle mass, subcutaneous fat (SFA), and BP were automatically measured using InBody 970 (InBody Co., Ltd., Seoul, Republic of Korea). The WC was measured using a tapeline at week 0, 4, and 8 visits. Blood samples were collected in serum-separating or ethylenediaminetetraacetic acid tubes after fasting overnight at the week 0 and 8 visits, separated into serum or plasma, and then stored below −80 °C before analysis.

### 2.7. Glycemic Control-Related Parameters and Serum Lipid Profiles

The fasting glucose levels in whole blood were measured using CareSens Dual (i-SENS, Inc., Seoul, Republic of Korea). Hemoglobin A1c (HbA1c, %) was measured using SD A1cCare (SD Biosensor, Inc., Suwon, Republic of Korea). The serum insulin and *C*-peptide levels were measured via the immunoassay method using a cobas e 801 analyzer (Roche Ltd., Mannheim, Germany). Homeostasis model assessment insulin resistance (HOMA-IR) was calculated as follows: HOMA-IR = [fasting insulin (μIU/mL) × fasting glucose (mg/dL)]/450. The serum levels of TG, total cholesterol (TC), and LDL-cholesterol (LDL-C) were measured using enzymatic assays on a Hitachi LABOSPECT 008AS automatic analyzer (Hitachi, Ltd., Tokyo, Japan). The serum HDL-C levels were measured using an enzymatic method after the precipitation of chylomicrons with dextran sulfate magnesium.

### 2.8. Statistical Analysis

IBM SPSS Statistics version 25.0 (IBM Corp., Armonk, NY, USA) was used for statistical analysis. The Wilcoxon signed-rank test (non-parametric paired *t*-test) was used to compare the results before and after the intervention. Between-group differences in baseline values and changes, respectively, were analyzed using the Mann–Whitney U test (non-parametric independent *t*-test). The Friedman test (non-parametric repeated measure analysis of variance) was used to compare the values at weeks 0, 4, and 8 of the intervention after adjusting for sex, age, cigarette smoking, and alcohol consumption. Partial correlation was also used with adjustment for sex and age. A *p*-value (P) of <0.05 was considered significant. In addition, reported *p*-values (P) are adjusted for relevant covariates as specified in each analysis. To control for multiple testing, the Benjamini–Hochberg false discovery rate (FDR) procedure was applied within predefined families of related comparisons (i.e., within each outcome variable for post hoc repeated-measures comparisons, within DAL-related subgroup comparisons, and within each time point for correlation analyses). Corresponding q-values (Q) (FDR-adjusted *p*-values) are reported. Statistical significance was defined as q-value < 0.05. All tests were two-sided. Given the exploratory nature of this single-arm study, findings are interpreted cautiously.

## 3. Results

### 3.1. Body Composition Parameters of the Study Participants After the 8-Week Intervention

**Figure 1:**
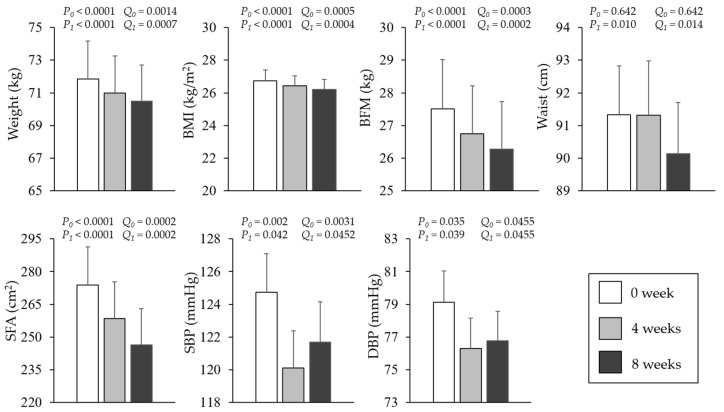
Anthropometric and glycemic parameters of participants during the intervention period. Means ± standard error. Tested via the Friedman test (non-parametric repeated-measures analysis of variance). Where applicable, analyses were adjusted for age, sex, cigarette smoking, and alcohol consumption. *P*_0_ indicates the comparison between week 0 and week 4; *P*_1_ indicates the comparison between week 0 and week 8. *Q*_0_ and *Q*_1_ denote the corresponding Benjamini–Hochberg false discovery rate (FDR)-adjusted q-values. Post hoc pairwise comparisons were conducted with adjustment for multiple testing using the FDR procedure across the entire predefined family of comparisons within this analysis. Covariate-adjusted *p*-values and FDR-adjusted q-values are reported. Statistical significance was defined as q < 0.05. BFM: body fat mass, BMI: body mass index, DBP: diastolic blood pressure, SBP: systolic blood pressure, SFA: subcutaneous fat area.

As shown in Figure 1, the intervention was associated with significant reductions in body weight, BMI, BFM, SFA, and both systolic and diastolic BPs, all of which remained significant after FDR correction (Q < 0.05). WC showed a significant decrease at 8 weeks but not at 4 weeks.

### 3.2. Changes in Dietary Acid Load Indices (NEAP and PRAL) During the 8-Week Intervention Period

**Figure 2:**
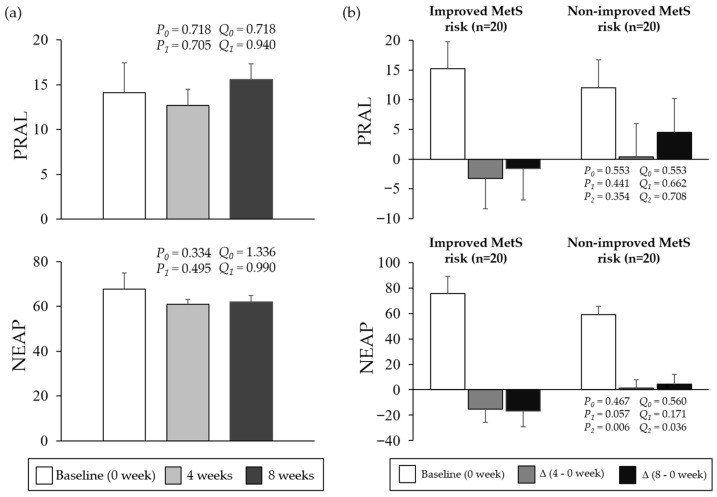
Changes in NEAP and PRAL scores during the 8-week intervention period. (**a**) Net endogenous acid production (NEAP) and potential renal acid load (PRAL) measured at baseline (week 0), week 4, and week 8 in all participants. Data are presented as means ± standard error (SE). Repeated-measures differences were evaluated using the Friedman test. *P*_0_ indicates the comparison between week 0 and week 4, and *P*_1_ indicates the comparison between week 0 and week 8. *Q*_0_ and *Q*_1_ denote the corresponding Benjamini–Hochberg false discovery rate (FDR)-adjusted q-values for *P*_0_ and *P*_1_. Where applicable, analyses were adjusted for age, sex, cigarette smoking, and alcohol consumption. To account for multiple testing, the FDR procedure was applied to the predefined family of comparisons. (**b**) Baseline values and changes in NEAP and PRAL from week 0 to week 4 and from week 0 to week 8, stratified by metabolic syndrome (MetS) risk improvement status at week 8. Participants were classified as improved if the number of MetS risk factors decreased after 8 weeks and as non-improved otherwise. Between-group comparisons were performed using the Mann–Whitney U test. *P*_0_ denotes the between-group comparison (improved vs. non-improved) of baseline NEAP and PRAL values. *P*_1_ and *P*_2_ denote between-group comparisons of changes from week 0 to week 4 and from week 0 to week 8, respectively. *Q*_0_, *Q*_1_, and *Q*_2_ denote the corresponding FDR-adjusted *q*-values for *P*_0_, *P*_1_, and *P*_2_. FDR correction was applied across all DAL-related subgroup comparisons in this panel. Reported *p*-values reflect covariate-adjusted analyses where applicable, and corresponding q-values represent FDR-adjusted *p*-values). Statistical significance was defined as q < 0.05.

Figure 2 illustrates changes in PRAL and NEAP during the 8-week intervention and comparisons stratified by MetS risk improvement status. Among all participants, neither PRAL nor NEAP showed a statistically significant change across time after FDR correction (all q > 0.05). In subgroup analyses, baseline values did not differ between the improved and non-improved MetS risk groups (q > 0.05). Although DAL indices tended to decrease in the improved group and increase in the non-improved group, only the between-group difference in ΔNEAP from week 0 to week 8 remained statistically significant after FDR correction (q < 0.05).

### 3.3. Changes in NQ and Nutrient Intake Following the 8-Week Intervention

Among all participants, the total NQ scores were significantly improved after the 8 weeks of intervention (51.0 ± 1.84 and 57.5 ± 1.62, *p* < 0.0001, Q = 0.0013), along with all of its components, except for diversity: balance (31.1 ± 2.76 and 35.9 ± 2.25, *p* = 0.013, Q = 0.0423), moderation (70.0 ± 2.29 and 77.0 ± 2.08, *p* = 0.007, Q = 0.0303), behavior (41.2 ± 2.75 and 52.9 ± 1.88, *p* < 0.0001, Q = 0.0007), and diversity (56.5 ± 2.55 and 60.0 ± 2.98, *p* = 0.178, Q = 0.4628). The mean daily energy and nutrient intakes did not differ significantly before and after the intervention. The energy intake and the proportions of macronutrients remained stable, and no significant changes were observed in the mineral intake levels (energy: 1530.9 ± 88.9 kcal and 1512.03 ± 7.505 kcal, *p* = 0.834, Q = 0.834; carbohydrate%: 54.32 ± 2.62, 51.32 ± 1.27, *p* = 0.273, Q = 0.4436; fat%: 26.22 ± 1.58, 28.45 ± 0.97, *p* = 0.225, Q = 0.4179; protein%: 17.29 ± 0.90, 17.65 ± 0.31, *p* = 0.214, Q = 0.4628; calcium 462.87 ± 48.28 mg, 453.01 ± 32.02 mg, *p* = 0.812, Q = 0.8797; phosphorus: 925.81 ± 64.93 mg, 967.75 ± 53.67 mg, *p* = 0.364, Q = 0.5258; potassium: 2097.3 ± 169.49 mg, 2137.82 ± 147.81 mg, *p* = 0.759, Q = 0.8970; magnesium: 87.18 ± 7.96 mg, 79.16 ± 5.96 mg, *p* = 0.426, Q = 0.5538).

### 3.4. Correlation Between DAL Scores and Anthropometric or Metabolic Parameters at Baseline

**Figure 3:**
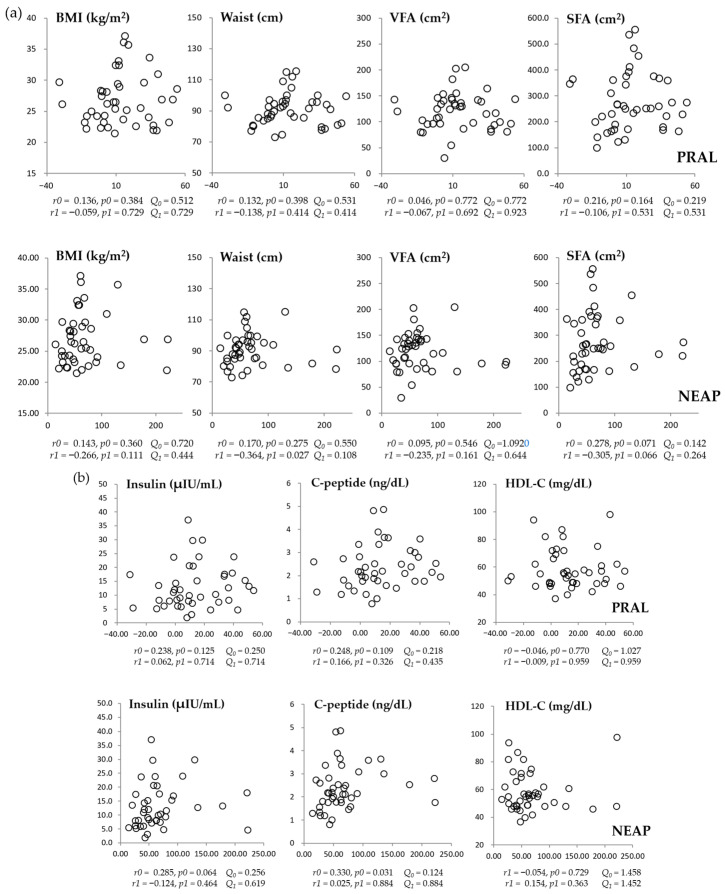
Correlations between dietary acid load scores (PRAL and NEAP) and anthropometric parameters (**a**) or metabolic biochemical measures (**b**) at baseline. *r*, correlation coefficient; *p*, *p*-value; *r0* and *p0*, tested by the Spearman correlation analysis (unadjusted); *r1* and *p1*, tested by the partial correlation analysis (adjusted for age and sex). *Q*_0_ and *Q*_1_ denote the corresponding the Benjamini–Hochberg false discovery rate (FDR)-adjusted q-values for *p0* and *p1*, respectively. To control for multiple comparisons, FDR correction was applied within each time point across all tested correlations. Both covariate-adjusted *p*-values and FDR-adjusted q-values are presented. Statistical significance was defined as q < 0.05. BMI, body mass index; HDL-C: high-density lipoprotein cholesterol; NEAP, net endogenous acid production; PRAL, potential renal acid load; SFA, subcutaneous fat area; VFA, visceral fat area.

At baseline, neither NEAP nor PRAL was significantly associated with anthropometric or metabolic parameters after FDR correction (Figure 3a,b). Although NEAP was inversely correlated with waist circumference before correction (adjusted *p* = 0.027), this association did not remain significant after FDR adjustment (q = 0.108).

### 3.5. Correlation Between DAL Scores and Anthropometric or Metabolic Parameters After the Intervention

**Figure 4:**
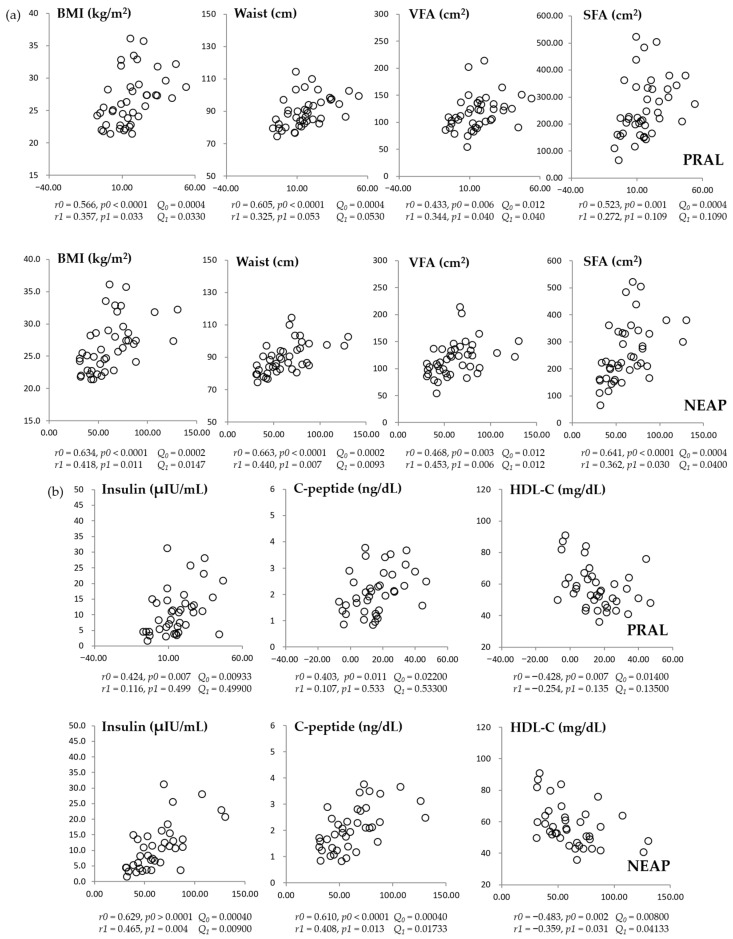
Correlations between dietary acid load scores (PRAL and NEAP) and anthropometric parameters (**a**) or metabolic measures (**b**) after 8 weeks of intervention. *r*, correlation coefficient; *p*, *p*-value; *r0* and *p0*, tested by the Spearman correlation analysis (unadjusted); *r1* and *p1*, tested by the partial correlation analysis (adjusted for age and sex). *Q*_0_ and *Q*_1_ denote the corresponding the Benjamini–Hochberg false discovery rate (FDR)-adjusted q-values for *p0* and *p1*, respectively. To control for multiple comparisons, FDR correction was applied within each time point across all tested correlations. Both covariate-adjusted *p*-values and FDR-adjusted q-values are presented. Statistical significance was defined as Q < 0.05. BMI, body mass index; HDL-C: high-density lipoprotein cholesterol; NEAP, net endogenous acid production; PRAL, potential renal acid load; SFA, subcutaneous fat area; VFA, visceral fat area.

After 8 weeks, both NEAP and PRAL were positively correlated with BMI, WC, VFA, and SFA (Figure 4a). These associations remained statistically significant after FDR correction for NEAP; however, for PRAL, the correlations with WC and SFA did not remain significant after correction. Regarding metabolic biochemical parameters (Figure 4b), significant associations at week 8 were observed when DAL was expressed as NEAP but not as PRAL after FDR correction. Although NEAP showed correlations with fasting glucose at baseline and with HOMA-IR at week 8 before correction, these associations did not remain statistically significant after FDR adjustment. Neither NEAP nor PRAL was significantly associated with other lipid profiles, including TG, LDL-C, and TC.

### 3.6. Correlations Between Changes in Dietary Acid Load Scores (PRAL and NEAP) and Changes in Anthropometric Parameters or Metabolic Measures from Baseline to 8 Weeks of Intervention

**Figure 5:**
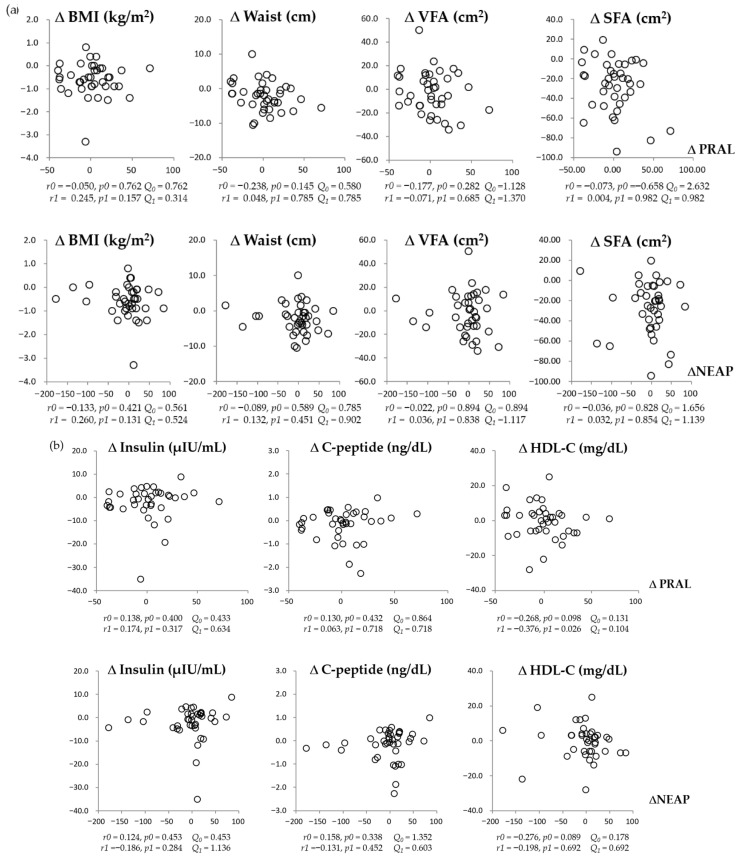
Correlations between changes in dietary acid load scores (PRAL and NEAP) and changes in anthropometric parameters (**a**) or metabolic measures (**b**) from baseline to 8 weeks of intervention. *r*, correlation coefficient; *p*, *p*-value; *r0* and *p0*, were derived from Spearman correlation analysis (unadjusted); *r1* and *p1*, from partial correlation analysis adjusted for age, sex, baseline BMI and baseline DAL values. *Q*_0_ and *Q*_1_ denote the corresponding the Benjamini–Hochberg false discovery rate (FDR)-adjusted q-values for *p0* and *p1*, respectively. To control for multiple comparisons, FDR correction was applied within each time point across all tested correlations. Both covariate-adjusted *p*-values and FDR-adjusted q-values are presented. Statistical significance was defined as q < 0.05. BMI, body mass index; HDL-C: high-density lipoprotein cholesterol; NEAP, net endogenous acid production; PRAL, potential renal acid load; SFA, subcutaneous fat area; VFA, visceral fat area.

Figure 5 shows no significant associations between 8-week changes (Δ) in DAL scores and anthropometric or metabolic parameters after FDR correction in the overall sample. In exploratory subgroup analyses stratified by the direction of NEAP change, a positive association between ΔNEAP and ΔBMI was observed within the NEAP-decrease subgroup (q < 0.05). A similar trend was observed in the PRAL-stratified analysis, although it did not remain statistically significant after FDR correction (q = 0.092). Given the exploratory and subgroup-based nature of these analyses, these findings should be interpreted cautiously.

## 4. Discussion

The present study extends the existing research on the DAL by evaluating its short-term responsiveness to eating habit modification within an intervention-based context. While prior investigations have predominantly focused on cross-sectional associations between DAL and cardiometabolic risk, our findings provide preliminary evidence that DAL measures, particularly the NEAP, may be associated with metabolic status following dietary modification. However, given that change-to-change associations were not statistically significant after FDR correction, these findings should be interpreted cautiously. By directly comparing the NEAP and PRAL and incorporating the NQ as an integrated dietary quality indicator, this study offers a more nuanced understanding of how DAL indices behave in real-world dietary modification settings.

Previous studies have demonstrated that chronic metabolic disorders including obesity, cardiovascular diseases, and pulmonary diseases are associated with DAL scores expressed as the NEAP or PRAL [20,21,22,24,25]. Women with higher NEAP scores showed higher levels of body weight, WC, and TG levels, and overweight/obese adolescents with higher PRAL and NEAP scores exhibited higher fasting glucose levels [21,26]. Elevated NEAP levels have been independently and synergistically associated with an increased risk of chronic obstructive pulmonary disease, regardless of smoking status. However, one study reported that the cardiovascular disease risk was significantly associated with the NEAP but not with the PRAL [27].

In this study, stratified analyses revealed distinct trajectories of DAL indices according to the MetS risk improvement status. Although no significant overall changes in the PRAL or NEAP were observed in the overall participants, those with improved MetS risk exhibited consistent reductions in both indices at weeks 4 and 8, whereas increases were observed in the non-improved group. Notably, the NEAP demonstrated a more consistent and pronounced reduction pattern in the improved group, and only the 8-week change in NEAP reached statistical significance in between-group comparisons.

These findings suggest that lower DAL levels, particularly when expressed as NEAP, were more consistently observed in participants who demonstrated metabolic improvement. However, given that change-to-change associations were not statistically significant after FDR correction, these observations should be interpreted cautiously and not as evidence of a causal effect. Given that the NEAP is derived primarily from protein and potassium intake, it may capture certain aspects of dietary modification differently from PRAL, which incorporates multiple mineral components. Thus, in this exploratory context, the NEAP may warrant further investigation as a potentially responsive index in short-term dietary intervention studies. Importantly, most previous studies examining DAL and MetS have been cross-sectional in nature. In contrast, the present study evaluated changes following eating habit modification, providing exploratory evidence that behavioral dietary intervention may be associated with changes in the DAL and metabolic parameters. However, given the quasi-experimental design, these findings should be interpreted as associative and hypothesis-generating.

Body composition parameters such as the BMI, body fat, SFA, and whole-blood glucose were significantly improved after the intervention. The BP and HbA1c levels were also significantly improved at week 4, and the insulin resistance decreased significantly at week 8. Correlations between the DAL scores (NEAP and PRAL) and MetS-related parameters, which were not significant at baseline, reached statistical significance after the intervention. Although no significant correlations were observed at baseline, cross-sectional associations between DAL indices and adiposity-related measures emerged at week 8. However, no significant change-to-change correlations were detected after FDR correction. This pattern suggests that DAL levels may be associated with adiposity status following dietary modification; however, the absence of significant longitudinal associations limits causal interpretation, and the results should therefore be considered exploratory. Overall, eating habit modification was accompanied by improvements in MetS-related parameters, while DAL indices exhibited differential patterns according to MetS risk improvement status. In addition, at least one month of customized education focusing on eating behavior modification was associated with improvements in NQ scores and overall metabolic parameters, alongside observable patterns in DAL indices.

In the present study, total NQ scores and several domain-specific scores improved significantly following the intervention, whereas total energy intake, macronutrient distribution, and micronutrients included in the DAL estimation did not show statistically significant changes at the group level. This apparent discrepancy may be explained by the conceptual differences between NQ and nutrient-based assessments. The NQ primarily reflects overall dietary quality and eating patterns, including food group balance, moderation of acidogenic foods, and health-related eating behaviors, rather than absolute nutrient quantities. Accordingly, improvements in NQ scores may indicate qualitative shifts in dietary patterns such as a relatively increased intake of fruits and vegetables and a reduced frequency of highly acid-producing foods even in the absence of measurable changes in the total nutrient intake. While such pattern-level modifications may not be fully captured by conventional nutrient analyses, they may partially correspond with variation in DAL indices, particularly NEAP, which is influenced by the balance between protein and potassium intake. These findings suggest that diet quality-based indices may capture behavioral and compositional dietary changes not readily detected through quantitative nutrient assessment alone. However, given the exploratory design and limited sample size, these interpretations should be considered hypothesis-generating. The absence of statistically significant nutrient changes may also reflect limited statistical power to detect small quantitative differences.

In a randomized clinical trial involving a 16-week plant-based dietary intervention, the PRAL and NEAP scores were positively correlated with the body weight and body fat [20]. Another study suggested that insulin resistance associated with impaired glucose homeostasis may be influenced by dietary patterns rich in alkaline vegetables and fruits [28]. Conversely, Westernized dietary patterns have been associated with higher triglyceride levels, elevated systolic BP, and increased blood glucose, along with reduced HDL-C levels, thereby contributing to increased cardiometabolic risk [29]. Animal-derived foods such as cheese, eggs, fish, meat, as well as refined grains contribute to higher acid production due to their content of sulfur-containing amino acids, phosphorus, and chloride. In contrast, fruits and vegetables, which contain relatively higher levels of glutamate and citrate, contribute to base production and help support acid-base balance [30]. Accordingly, dietary patterns characterized by a high intake of acid-producing foods and a low intake of alkaline foods may increase endogenous acid production and, consequently, elevate the DAL scores. Chronic exposure to higher DAL has been proposed to contribute to low-grade metabolic disturbances, including hypertension and insulin resistance [13,31,32]. In healthy individuals, acid-base homeostasis is maintained through buffering systems in the blood, respiration, and renal excretion.

The present findings indicate that eating habit modification was accompanied by differential patterns in DAL scores and concurrent improvements in anthropometric and metabolic parameters. While causality cannot be inferred, these observations raise the possibility that dietary patterns lower in acid-producing foods and richer in vegetables and fruits may be associated with more favorable metabolic profiles.

Understanding the short-term behavior of DAL indices has potential clinical relevance. If DAL measures can reflect aspects of dietary pattern modification within relatively brief intervention period, they may serve as practical monitoring tools in lifestyle-based cardiometabolic risk management. In populations undergoing rapid dietary transitions, such as those observed in East Asia, identifying modifiable dietary constructs that respond to behavioral guidance is particularly important. Although the present findings should be interpreted cautiously due to the exploratory design and limited sample size, they suggest that DAL indices may warrant further investigation as potential intermediate markers in studies examining the relationship between dietary modification and metabolic risk. However, this study has several limitations. First, the single-arm quasi-experimental pre–post study without a control group limits internal validity, thereby constraining causal interpretation. Although within-subject comparisons reduce inter-individual variability, alternative explanations such as regression to the mean, seasonal variation, temporal trends, or increased health awareness during study participation cannot be excluded. Therefore, the observed associations between changes in the DAL and MetS-related parameters should not be interpreted as evidence of causality. Second, the dietary intake was assessed using self-reported food diaries, which were completed according to standardized instructions and subsequently reviewed by a registered dietitian to enhance accuracy and completeness. However, self-reported dietary assessment methods remain inherently susceptible to recall and reporting bias. Furthermore, despite instructions to maintain habitual dietary patterns aside from the eating habit modification guidance, unmeasured changes in total energy intake or macronutrient composition may have partially contributed to the observed metabolic improvement. Future studies incorporating objective biomarkers of acid–base balance (e.g., urinary pH or net acid excretion) would strengthen the validity of DAL assessment. Third, the relatively short intervention period (8 weeks) limits conclusions regarding the long-term sustainability of observed patterns. Whether these associations persist over extended follow-up remains unknown. Fourth, as an exploratory investigation, no a priori sample size calculation was performed, and the study was not powered for definitive hypothesis testing. The relatively small sample size, combined with multiple statistical comparisons anthropometric, glycemic, lipid, and dietary variables, may have reduced statistical power, particularly for adjusted and longitudinal analyses. Although FDR correction was applied, the risks of both type I and type II error cannot be excluded. In addition, the predominance of female participants (~81%) may also limit the generalizability. Although sex was included as a covariate, detailed information on female hormonal status (e.g., menstrual phase or menopausal status) was not collected. Given that hormonal fluctuations may influence metabolic parameters, residual confounding cannot be ruled out. Accordingly, the present findings should be interpreted cautiously and regarded as preliminary and hypothesis-generating, warranting confirmation in adequately powered randomized controlled trials with appropriate comparator groups. Despite these limitations, the within-subject pre–post design reduces inter-individual variability and may have enhance sensitivity for detecting short-term within-person changes.

## 5. Conclusions

In this exploratory single-arm pre–post intervention study, differential patterns in DAL indices, particularly NEAP, were observed alongside favorable changes in anthropometric and metabolic parameters. Given the absence of a control group and the lack of significant longitudinal associations, these findings should not be interpreted as evidence of causality but rather as hypothesis-generating observations. Future randomized controlled trials are required to determine whether modification of DAL independently contributes to metabolic improvement.

## Data Availability

The data supporting the conclusions of this article will be available from the corresponding author upon reasonable request.
